# Changes in plasma ghrelin and leptin levels in patients with peptic ulcer and gastritis following eradication of *Helicobacter pylori* infection

**DOI:** 10.1186/s12876-016-0532-2

**Published:** 2016-10-04

**Authors:** Chika Kasai, Kazushi Sugimoto, Isao Moritani, Junichiro Tanaka, Yumi Oya, Hidekazu Inoue, Masahiko Tameda, Katsuya Shiraki, Masaaki Ito, Yoshiyuki Takei, Kojiro Takase

**Affiliations:** 1 Department of Gastroenterology, Mie Prefectural General Medical Center, Yokkaichi, Japan; 2 Department of Molecular and Laboratory Medicine, Mie University School of Medicine, 2-174 Edobashi, Tsu, Mie 514-8507 Japan; 3 Department of Gastroenterology and Hepatology, Mie University School of Medicine, Tsu, Japan; 4 Department of Cardiology and Nephrology, Mie University School of Medicine, Tsu, Japan

**Keywords:** Plasma Ghrelin and Leptin, *Helicobacter pylori*, Eradication

## Abstract

**Background:**

*Helicobacter pylori* (*H. pylori*) infection and eradication therapy have been known to influence gastric ghrelin and leptin secretion, which may lead to weight gain. However, the exact relationship between plasma ghrelin/leptin levels and *H. pylori* infection has remained controversial. The aim of this study was to investigate plasma ghrelin and leptin levels in *H. pylori*-positive and -negative patients, to compare the two levels of the hormones before and after *H. pylori* eradication, and to examine the correlation between body mass index (BMI) and active ghrelin or leptin levels, as well as that between atrophic pattern and active ghrelin or leptin levels.

**Methods:**

Seventy-two *H. pylori*-positive patients who underwent upper gastrointestinal endoscopy, 46 diagnosed as having peptic ulcer and 26 as atrophic gastritis, were enrolled. Control samples were obtained from 15 healthy *H. pylori*-negative volunteers. The extent of atrophic change of the gastric mucosa was assessed endoscopically. Body weight was measured and blood was collected before and 12 weeks after *H. pylori* eradication therapy. Blood samples were taken between 8 and 10 AM after an overnight fast.

**Results:**

Plasma ghrelin levels were significantly lower in *H. pylori*-positive patients than in *H. pylori*-negative patients. In particular, plasma active ghrelin levels were significantly lower in patients with gastritis compared with patients with peptic ulcer. Plasma ghrelin levels decreased after *H. pylori* eradication in both peptic ulcer and gastritis patients, while plasma leptin levels increased only in peptic ulcer patients. Plasma leptin levels and BMI were positively correlated, and active ghrelin levels and atrophic pattern were weakly negatively correlated in peptic ulcer patients.

**Conclusion:**

*H. pylori* infection and eradication therapy may affect circulating ghrelin/leptin levels. This finding suggests a relationship between gastric mucosal injury induced by *H. pylori* infection and changes in plasma ghrelin and leptin levels.

## Background

*Helicobacter pylori* (*H. pylori*), a Gram-negative, spiral-shaped bacterium that colonizes the stomach, is a major cause of atrophic and chronic gastritis, peptic ulcers, and gastric malignant lesions such as mucosa-associated lymphoid tissue lymphoma and adenocarcinoma [1–3]. Eradication of *H. pylori* reduces the relapse rate of peptic ulcer [4] and the incidence of gastric cancer [5]. However, much attention has recently been paid to the inverse relationship of *H. pylori* infection and obesity [6, 7].

Appetite and energy expenditure are regulated mainly by two hormones, ghrelin and leptin, produced in the gastric mucosa, which may be modified by *H. pylori* colonization [8]. Ghrelin, a 28-amino acid, novel appetite-stimulating peptide produced predominantly by the stomach, is thought to be a strong growth hormone releaser [9]. Ghrelin exists in two different forms: acylated ghrelin, octanoylated, in serine3 (active ghrelin), and desacyl-ghrelin, without the octanoyl group [10]. Active ghrelin has a short half-life, and once released, it will be subsequently converted to desacyl-ghrelin [11]. Activation of ghrelin occurs via the enzyme ghrelin O-acyltransferase (GOAT) which is responsible for adding an N-octanoylated serine in potion 3 to the proghrelin peptide [12]. Desacyl-ghrelin is notably less potent on the GHS-receptor than active ghrelin [13]. It is generally known that these two forms of ghrelin have differential effects in the tissues. While active ghrelin has been implicated in the control of food intake and shown to evoke weight gain by actions in the hypothalamus [14, 15], desacyl-ghrelin is thought to be also involved in energy balance in some way, but its exact role is unknown. On the other hand, leptin is a 16-kDa protein, a product of the Ob gene, secreted primarily from adipose tissue cells [16]. It has recently been found that leptin is also present in gastric mucosa [17–19]. This hormone plays a role of mediator in the long-term regulation of energy balance, suppressing food intake, and thereby inducing weight loss [8, 20].

A number of studies have reported the relationship between plasma ghrelin/leptin levels and the effects of *H. pylori* infection and eradication. A study by Nwokolo et al., first reporting on the possible relationship between ghrelin and the effect of *H. pylori* eradication, showed that cure of *H. pylori* increased plasma ghrelin levels in healthy asymptomatic subjects, which in turn may lead to increased appetite and weight gain [21]. In contrast, some studies reported that plasma ghrelin levels decreased following *H. pylori* eradication [22, 23]. Nonetheless, a number of other studies showed that *H. pylori* infection and/or eradication therapy had no effect on ghrelin levels [24, 25] and leptin levels [26]. In addition, Azuma et al. reported that gastric leptin expression was significantly increased by *H. pylori* infection, and gastric leptin expression was reduced after *H. pylori* eradication, but serum leptin levels did not change significantly after cure of *H. pylori* infection [27]. It has been thought that the relationships between *H. pylori* and production of these hormones are regulated by the *H. pylori* strain [28], the extent of atrophic gastritis induced by *H. pylori* infection [29], the duration of follow-up, and other unexplained factors. However, the exact mechanism by which *H. pylori* eradication may interface with plasma ghrelin and leptin to affect body weight has remained unknown.

Therefore, in the present study, plasma ghrelin and leptin levels were measured in *H. pylori-*positive and -negative patients, and the levels of the two hormones were compared before and after *H. pylori* eradication. Furthermore, the correlations between body mass index (BMI) and active ghrelin or leptin levels and between atrophic pattern and active ghrelin or leptin levels were also examined.

## Methods

### Human subjects

The study subjects were 72 *H. pylori*-positive patients referred for upper gastrointestinal endoscopy at Mie Prefectural General Medical Center, Yokkaichi, Japan, between November 2011 and October 2014. The subjects’ diagnoses were duodenal and gastric ulcer in 46 and atrophic gastritis in 26. Control samples were obtained from 15 healthy *H. pylori*-negative volunteers. All patients and controls received an explanation of the procedures and possible risks associated with the study and gave their written, informed consent to participate. This study was performed in conformity with the Declaration of Helsinki and was approved by our institutional ethics committee (authorization number 2011-4, Mie Prefectural General Medical Center, Yokkaichi, Japan). The exclusion criteria were pregnancy, BMI >30 kg/m^2^, diabetes mellitus, cachectic state including advanced cancer, systemic infection, thyroid and liver disease, renal impairment, use of medications effective against *H. pylori* during the preceding 3 months, history of eradication therapy before the study, and history of previous gastric surgery.

### Eradication therapy and data collection

*H. pylori*-positive patients received triple therapy with lansoprazole 30 mg, amoxicillin 750 mg, and clarithromycin 200 mg twice per day for 7 days after the endoscopic examination. Body weight was measured and blood was collected before and 12 weeks after the treatment. Blood samples were taken between 8 and 10 AM after an overnight fast, transferred into BD™ P800 tubes (Becton-Dickinson, Franklin Lakes, NJ) containing spray-dried K_2_EDTA anticoagulant and a proprietary cocktail of protease, esterase, and dipeptidyl peptidase 4 (DPP-4) inhibitors. The collected samples were centrifuged at 1100–1300 × g for 10 min, and plasma was separated and stored at -80 °C until assay.

### Endoscopic diagnosis of atrophic gastritis

Endoscopic diagnosis of gastric mucosal atrophy was performed using the Kimura-Takemoto classification of atrophic pattern [30]. According to this classification, gastric mucosal atrophy is classified as closed-type if an atrophic boundary exists between the fundic mucosa and the pyloric mucosa in the antrum or lesser curvature of the gastric body. In the C-0 type, there are no atrophic changes. In the C-1 type, there are atrophic changes visible only in the antrum. In the C-2 type, the atrophic borders exist on the lesser curvature of the lower portion of the stomach body. In the C-3 type, atrophic borders are found on the lesser curvature of the upper portion of the stomach body. On the other hand, atrophy is classified as open type if an atrophic border lies in the lateral wall or greater curvature of the gastric body. In the O-1 type, atrophic borders are found between the lesser curvature and the lateral wall of the gastric body. In the O-2 type, atrophic changes are spread along the lateral wall. In the O-3 type, a border exists between the lateral wall and the greater curvature.

### Schedule of examinations for *H. pylori* infection

Endoscopic examination and determination of *H. pylori* infection were performed by RUT (Pyloritek, Serim Laboratories, Elkhart, IN) before treatment. The success of the *H. pylori* eradication therapy was assessed by ImmunoCard STAT!®HpSA®Stool antigen test (Meridian Bioscience Inc., Cincinnati, OH) 12 weeks after the cessation of therapy.

### Hormone assays

ELISA kits were used for the measurement of active serum ghrelin (SCETI K.K., Tokyo, Japan), des-acyl serum ghrelin (SCETI K.K.), and leptin (Cosmic Corporation, Tokyo, Japan). Serum levels of both ghrelin and leptin were measured and calculated according to the manufacturer instructions. Ghrelin levels are expressed as fmol/mL and leptin levels as ng/ml.

### Statistical analysis

Continuous variables were compared using the Kruskal-Wallis test or the Mann-Whitney test (two-sided), and categorical variables were compared using Fisher’s exact test. Paired measures of plasma ghrelin and leptin levels were analyzed using the Wilcoxon signed-rank test. Ghrelin/leptin levels and BMI or atrophic pattern were assessed for correlations with Pearson correlation coefficients. All statistical analyses were performed with IBM SPSS software Ver. 22. Data are expressed as mean ± SD. *P* values less than 0.05 were considered significant.

## Results

### Baseline characteristics

The characteristics of the subjects are shown in Table 1. A total of 72 *H. pylori*-positive patients (46 peptic ulcer and 26 atrophic gastritis) and 15 healthy *H. pylori*-negative volunteers were studied. There were no significant differences in BMI between each pair of groups. They included 20 current smokers and 31 alcohol drinkers. There were no differences in laboratory data (data not shown). *H. pylori* eradication was successful in 39 of 46 peptic ulcer patients and 18 of 26 atrophic gastritis patients who received eradication therapy.

**Table 1:** Demographic and clinical characteristics of the study groups

	Control	Peptic Ulcer	Chronic Gastritis	*P* value
	*N* = 15	*N* = 46	*N* = 26	
Age	45.9 ± 9.3	53.0 ± 15.6	60.8 ± 11.7	0.001
Gender, M: *n* (%)	6 (40 %)	32 (69.5 %)	10 (38.5 %)	0.017
BMI (kg/m^2^)	21.7 ± 2.2	23.0 ± 3.4	22.1 ± 2.9	0.260

*P* values are based on Kruskal-Wallis test or the Mann-Whitney test (two-sided) for continuous variables and Fisher’ exact test for categorical variable

*BMI* body mass index

### Ghrelin and leptin levels at the initial assessment

**Fig. 1:**
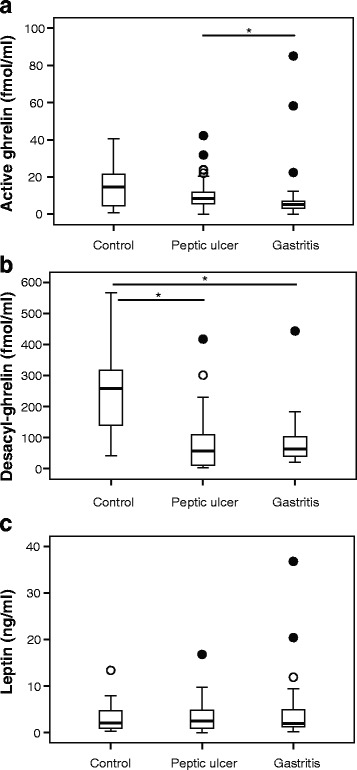
**a** Active plasma ghrelin, **b** desacyl-ghrelin, and **c** leptin levels of control, peptic ulcer, and gastritis patients at the initial assessment *P < 0.05

Plasma active ghrelin levels were significantly lower in patients with gastritis than in patients with peptic ulcer (Fig. 1a). Notably, plasma desacyl-ghrelin levels were significantly higher in *H. pylori*-negative control patients than in *H. pylori*-positive patients (both peptic ulcer and gastritis) (Fig. 1b). With regard to plasma leptin levels, no significant differences were found between each pair of three groups (Fig. 1c). Since plasma ghrelin levels were affected by the presence or absence of *H. pylori*, whether *H. pylori* eradication affects BMI and circulating ghrelin/leptin levels, as well as whether the treatment regulates the levels of these two hormones to those of *H. pylori*-negative patients, was evaluated.

### Changes in BMI after *H. pylori* eradication

There were no significant changes in BMI in after *H. pylori* eradication in both peptic ulcer and gastritis groups, regardless of its success (data not shown).

### Ghrelin and leptin levels at the initial and 12^th^ week assessment

**Fig. 2:**
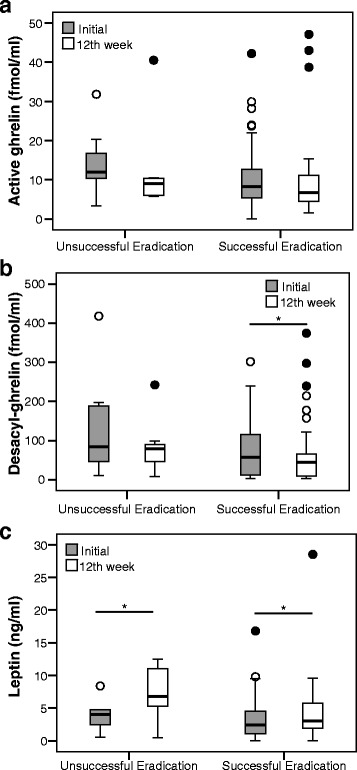
Changes in each hormone in peptic ulcer patients at the initial and 12-week after *H. pylori* eradication. **a** Active ghrelin levels in unsuccessful eradication group (14.5 ± 9.2 vs. 12.6 ± 12.5; *P* = 0.398), and successful eradication group (10.9 ± 99.7 vs. 9.9 ± 10.3; *P* = 0.083). **b** Desacyl-ghrelin levels in unsuccessful eradication group (140.7 ± 141.6 vs. 86.1 ± 75.2; *P* = 0.063), and successful eradication group (76.7 ± 77.3 vs. 68.2 ± 84.7; *P* = 0.043). **c** Leptin levels in unsuccessful eradication group (4.0 ± 2.6 vs. 7.1 ± 4.3; *P* = 0.046), and successful eradication group (3.4 ± 3.6 vs.4.4 ± 5.0; *P* = 0.006) *P < 0.05

**Fig. 3:**
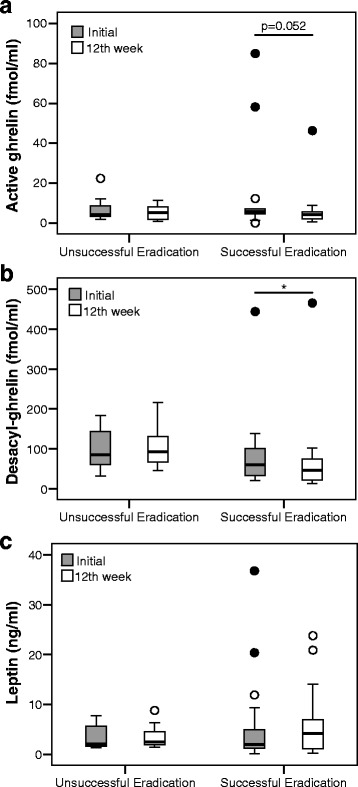
Changes in each hormone in gastritis patients at the initial and 12-week after *H. pylori* eradication. **a** Active ghrelin levels in unsuccessful eradication group (7.1 ± 6.9 vs. 5.4 ± 4.0; *P* = 0.499), and successful eradication group (12.9 ± 22.8 vs. 6.8 ± 10.5; *P* = 0.052). **b** Desacyl-ghrelin levels in unsuccessful eradication group (99.1 ± 56.0 vs. 105.2 ± 55.2; *P* = 0.123), and successful eradication group (86.4 ± 99.7 vs. 73.8 ± 104.9; *P* = 0.022). **c** Leptin levels in unsuccessful eradication group (3.5 ± 12.6 vs. 3.5 ± 2.6; *P* = 0.483), and successful eradication group (6.2 ± 9.5 vs. 6.2 ± 7.2; *P* = 0.097) *P < 0.05

Whereas significant changes in BMI were not found in patients after successful *H. pylori* eradication, altered levels of circulating ghrelin and leptin were observed. In peptic ulcer patients, although not significant in all cases, ghrelin levels decreased after *H. pylori* eradication (Fig. 2a and b). There was a significant increase in leptin levels in both successfully and unsuccessfully eradicated patients (Fig. 2c). In the case of gastritis patients, there was a significant decrease in ghrelin levels in eradicated patients (Fig. 3a and b), while no changes were found in leptin levels (Fig. 3c).

### Relationships between BMI and active ghrelin or leptin levels

**Fig. 4:**
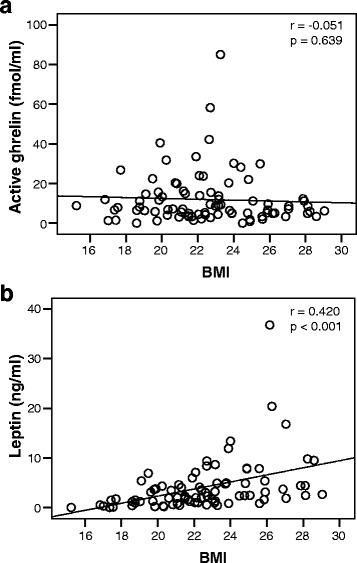
The relationship between BMI and the initial plasma active ghrelin (**a**) / leptin (**b**) levels. The initial plasma leptin levels and BMI are positively correlated (*r* = 0.420, *P* < 0.001)

Although the initial active ghrelin levels and BMI were not correlated, the initial plasma leptin levels and BMI were positively correlated (*r* = 0.420, *P* < 0.001) (Fig. 4). This correlation might be attributed to the fact that leptin is secreted primarily from adipose tissue cells.

### Relationships between atrophic pattern and active ghrelin or leptin levels

**Fig. 5:**
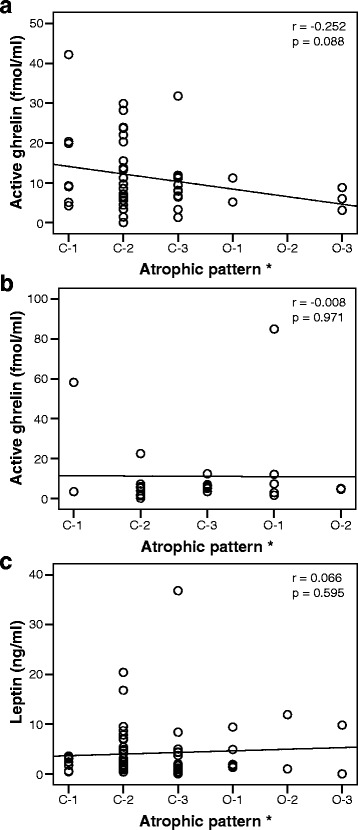
The relationship between atrophic pattern and the initial plasma active ghrelin (**a**: peptic ulcer, **b**: gastritis) / leptin (**c**) levels

Although not significant, the initial active ghrelin levels showed a weak negative correlation with atrophic pattern in peptic ulcer patients, but no correlation in gastritis patients (Fig. 5a and b). The initial leptin levels showed no correlation with atrophic pattern in peptic ulcer and gastritis patients (Fig. 5c).

## Discussion

In the current study, there were three major findings. (i) Plasma ghrelin levels were significantly lower in *H. pylori*-positive patients than in *H. pylori*-negative control volunteers. In particular, plasma active ghrelin levels were significantly lower in patients with gastritis than in patients with peptic ulcer. (ii) Plasma active and desacyl ghrelin levels decreased after *H. pylori* eradication in both peptic ulcer and gastritis patients, while plasma leptin levels increased only in peptic ulcer patients. (iii) Plasma leptin levels and BMI were positively correlated, and the active ghrelin levels and atrophic pattern were weakly negatively correlated in peptic ulcer patients.

In common with previous studies [29, 31–33], plasma ghrelin levels were significantly lower in *H. pylori*-positive patients than in *H. pylori*-negative control volunteers. It seems logical that *H. pylori*-positive patients with atrophic gastric mucosa have low plasma ghrelin levels, considering the negative correlation between active ghrelin levels and atrophic pattern, as also proposed by Kawashima et al [29]. In addition, Isomoto et al. reported that *H. pylori*-positive patients with type I strain (expressing the virulence factors of cytotoxin-associated gene-A and Vacuolating cytotoxin A) have lower circulating ghrelin levels than those with the less virulent type II strain (expressing no virulence factors) [28]. The population analyzed in the present study was a Japanese population with Type I strain (a known contributor to a higher incidence of gastric cancer) [34], hence the lower plasma ghrelin levels in *H. pylori*-positive patients than in *H. pylori*-negative patients.

Furthermore, consistent with Suzuki et al.[35] and Isomoto et al.[36], plasma ghrelin levels were significantly lower in patients with gastritis than in those with peptic ulcer. A recent study suggested that plasma ghrelin levels increase in response to severe gastric mucosal oxidative stress induced by acute gastritis and peptic ulcer [37]. These results suggested that long duration of *H. pylori* infection reduced circulating ghrelin levels, presumably by destroying ghrelin-producing cells, and that acute gastric mucosal injury such as peptic ulcer increased circulating ghrelin levels because of stress related to ghrelin secretion.

The effect of *H. pylori* eradication on circulating ghrelin/leptin levels has remained controversial. Nweneka et al. reviewed previous studies on circulating ghrelin levels before and after *H. pylori* eradication in a meta-analysis [38]. Some studies reported that there was no significant difference in circulating ghrelin levels after *H. pylori* eradication [24, 31, 39], while others reported an increase [21, 29, 40] or a decrease [22, 23]. They concluded that *H. pylori* eradication did not have any significant effect on circulating ghrelin levels. On the other hand, Bocian et al. showed that *H. pylori* eradication was associated with increased circulating leptin levels and decreased ghrelin levels, thereby resulting in an increased BMI in many studies [7]. Consistent with Bocian et al. [7], the present results showed that plasma ghrelin levels decreased after *H. pylori* eradication in both peptic ulcer and gastritis patients, and plasma leptin levels increased in peptic ulcer patients. A possible mechanism for decreased levels of plasma ghrelin after *H. pylori* eradication is as follows: before eradication, plasma ghrelin is released transiently into blood due to gastric mucosal oxidative stress and injury induced by *H. pylori* infection, and after eradication, the release of plasma ghrelin decreases in accordance with improvement of gastric mucosal injury. The decreased ghrelin levels in the present study are possibly attributed to the relatively short term of observation and it can be further assumed that the ghrelin levels would return to normal or even higher in a longer-term follow-up observation.

The increase of plasma leptin levels after eradication in peptic ulcer patients needs explanation. Plasma leptin levels and BMI were positively correlated, while there were no significant changes in BMI after eradication in the present study. Previous studies suggested that short-term fasting caused a decline in circulating leptin concentration much greater than the change in adipose mass [41, 42]. Regardless of the success or failure of *H. pylori* eradication, presumably, the suppressed appetite of peptic ulcer patients improved with the healing of the ulcer, which led to increased food intake and increased plasma leptin levels as a consequence. In contrast, gastritis patients did not show a reduced appetite in the first place. That is probably the reason they exhibited changes neither in dietary behavior, nor in the concomitant plasma leptin levels. We suspect that there is a complex relationship among leptin secretion, adipose tissue mass, and gastric mucosal injury.

Remarkably, in the present study, both ghrelin and leptin levels before/after *H.pylori* eradication were measured in relation to peptic ulcer and gastritis. The most important findings are that our study showed the levels of the hormones changed independently and that the changes differed in peptic ulcer and gastritis patients.

The present study had certain limitations: lack of histological evaluation such as ghrelin/leptin expression in the stomach, small sample size, and short-duration of follow-up. Therefore, we believe further research including histological evaluation will definitely improve our understanding in a future long-term study.

## Conclusion

In conclusion, the results of the current study showed that plasma ghrelin levels were significantly lower in *H. pylori*-positive patients than in *H. pylori*-negative patients. Furthermore, plasma active and desacyl ghrelin levels decreased and plasma leptin levels increased after *H. pylori* eradication. However, the precise mechanism remains an open question for research. Further study will be necessary to elucidate the exact effect of *H. pylori* eradication on plasma ghrelin/leptin levels, which may lead to body weight gain and the ultimate development of lifestyle-related diseases.

## Abbreviations

BMI: Body mass index
*H. pylori*: *Helicobacter pylori*
